# Vaccine hesitancy and post-vaccination adherence to safety measures: A mixed-method study

**DOI:** 10.3389/fpubh.2023.1072740

**Published:** 2023-03-31

**Authors:** Ayesha Inam, Asia Mushtaq, Sahira Zaman, Samia Wasif, Mah Noor, Hania Asghar Khan

**Affiliations:** ^1^Department of Humanities, COMSATS University Islamabad, Islamabad, Pakistan; ^2^Department of Applied Psychology, National University of Modern Languages, Islamabad, Pakistan; ^3^Department of Gender Studies, Fatima Jinnah Women University Rawalpindi, Rawalpindi, Pakistan

**Keywords:** COVID-19, vaccine hesitancy, protection motivation theory, vaccination behavior, safety measures, Pakistan

## Abstract

**Background:**

Despite being recognized as one of the most successful public health measures, vaccination is still considered to be unnecessary and unreliable in the context of the COVID-19 pandemic. The current study utilized a two-pronged approach in analyzing vaccine hesitancy and health behaviors after vaccination by employing a mixed-method design. Phase 1 was aimed at identifying predictors of COVID-19 vaccine hesitancy and acceptance among the Pakistani population using protection motivation theory (PMT), whereas Phase 2 was aimed at exploring the factors related to the vaccination of COVID-19.

**Method:**

A convenient sample of 1,736 individuals from the vaccine-eligible population (12 years and above) was selected to collect data on vaccine hesitancy and acceptance (Phase 1). Phase 2 of the study explored post-vaccination health behaviors, especially adherence to safety measures for COVID-19, through 23 in-depth interviews with the vaccinated population.

**Results:**

Multiple regression analyses showed that response cost is a major predictor of vaccine hesitancy (in Phase 1). In terms of the role of demographic variables, the results showed that being male (for severity: B = −0.481; threat appraisal: B = −0.737), old age (B = −0.044), not vaccinated, and not infected with COVID-19 (themselves and family members) are strongly associated with vaccination hesitancy. Results of thematic analysis in Phase 2 revealed that perceived individual experience and insensitivity toward the severity of the disease are strongly associated with a lack of adherence to safety measures of COVID-19. Faith and religious beliefs and reliance on traditional remedies are also key predictors of people's general non-compliance to health behaviors. One interesting aspect that was revealed in the analysis was the general financially and socially destabilized situation in the context of developing countries that contributed to general apathy in the pandemic situation.

**Conclusion:**

The findings of the current study may help in devising a health model for the public from the developing world to deal with future pandemic situations.

## 1. Introduction

Vaccine hesitancy refers to the delay of acceptance or complete refusal of vaccine administration despite its free-of-cost availability to the public (1). People who hesitate to take vaccines are a heterogeneous group of individuals who usually fall on the continuum of complete acceptance and rejection which means they can refuse some vaccines and may accept few others (2). Therefore, the concept of vaccine hesitancy is multi-dimensional because its determinants are context-specific, which varies across time, place, and type of vaccines, influenced by socioeconomic (3), political, religiocultural, and scientific reasons (4), and increase the complexity of the decision-making about rejection or acceptance of vaccines (1). The determinants of vaccine hesitancy include confidence, complacency, and convenience toward vaccination (1). The contextual factor of hesitancy toward any vaccination program is also determined by historical, personal, and sociocultural factors; confidence in the country's health system; and risk/benefits attached to the vaccine (5). Education (6), poor communication (7), gender, minority groups, socioeconomic status, and information-seeking pattern (8) all impact confidence on vaccines and, conversely, hesitancy.

The World Health Organization (WHO) mentioned vaccine hesitancy as one of the top 10 threats to global health (9). The global research on coronavirus disease 2019 (COVID-19) vaccine hesitancy also showed mixed evidence of acceptance and reluctance rates. For example, this vaccination drive faced issues in the acceptability, reluctance, and hesitancy in different populations, such as in Portugal (10), Indonesia (11, 12), China (13–15), India (16), the United Kingdom, Ireland (17), and Italy (18). Psychological constructs of personality, altruism, religiosity, and internal locus of control were also essential indicators of COVID-19 vaccine hesitancy in the UK and Irish populations (19). On the contrary, in the Japanese population, people of older age groups, people living in rural areas, people with some medical conditions, and men showed more acceptance toward the COVID-19 vaccine (20). Vaccine literacy also contributes to building positive attitudes toward vaccine acceptance. An adequately informed public has reduced anxiety, improved behavior, and reduced disease transmission of COVID-19 (21). Similarly, vaccine efficacy is also an important indicator of hesitancy or acceptance of the COVID-19 vaccine in Southeast Asian countries (11). During the peak of the pandemic period, people were more favorable toward vaccine acceptance (22). Therefore, many factors influence people's attitudes toward approving or rejecting vaccines, especially COVID-19, in different socioeconomic, geographical, and demographic variables.

### 1.1. Pakistani context of vaccine hesitancy and adherence to safety

Pakistan has a history of reluctance toward all vaccines, and a prominent example of this attitude shows the plight of polio vaccination in the country (23). Several vaccines available for children in the Expanded Program on Immunization (EPI) showed a fall in the acceptance rate in Pakistan between 2015 and 2019 (8). Confidence fell in the importance, safety, and effectiveness of vaccines. Confidence was the strongest indicator of vaccine hesitancy compared to safety or efficacy, and religious beliefs also played an important role (8). The case of COVID-19 vaccination shows similar trends. In Pakistan, conspiracy beliefs, acceptability, preference, and willingness to pay are common factors for COVID-19 vaccine hesitancy (24). Yasmin et al. (25) also reported that more than half of the participants in her study were unsure of the safety (50%) and efficacy (51%) of the COVID-19 vaccine, whereas 42% were concerned about its side effects and 72% of the respondents planned to get vaccinated, whereas 28% refused to do so. Similarly, a meta-analysis of eight studies by Khalid et al. (26), including Arshad's and Yasmin's studies, reported that conspiracy beliefs, vaccine availability, healthcare system, religious matters, vaccine literacy, side effects, perceived fear, and natural immunity philosophy served as vaccine hesitancy indicators in Pakistan. Although a few of these studies included larger populations with diverse demographical backgrounds for generalizing the results of COVID-19 vaccine hesitancy, safety, and effectiveness in Pakistan, they only focused on quantitative data sets. However, no study to date has focused on post-vaccination adherence to safety measures. Therefore, this article aimed to fill the gap by focusing on larger quantitative data to measure vaccine hesitancy and also aimed to obtain in-depth information from respondents using a qualitative approach through in-depth interviews for post-vaccination adherence and safety of individuals. Thus, the generalization of the results could be performed with a certain confidence by converging the findings of both qualitative and quantitative data.

### 1.2. Protection motivation theory

Current research utilized the model of protection motivation theory (PMT). It is a widely used model in the health sector to understand and reflect on the attitudes and practices that motivate an individual toward performing protective behaviors. Rogers developed PMT in 1975 and it focuses on people's attitudes and behaviors in fear-instigating situations. According to this theory, threat appraisal and coping appraisal are two main components that describe a person's attitude toward fear-instigating situations. Threat appraisal is further explained with the help of four constructs: severity of the threat, vulnerability toward that threat, internal reward, and external reward. Coping appraisal comprises self-efficacy, response efficacy, and response cost. The theory explains that factors of severity, vulnerability, reward, self-efficacy, and response efficacy are associated with adaptive behaviors while response cost is related to maladaptive behaviors (27, 28). PMT has been tested in more than 30,000 studies. Applied in the context of the COVID-19 pandemic, Okuhara et al. (29) showed that perceived severity and self-efficacy significantly correlated with staying-at-home behavior during the first wave of the pandemic in Japan. Farooq et al. (30) also concluded that perceived severity and self-efficacy significantly correlated with the intention to self-isolate, but response cost negatively affected this intention. Literature on PMT application on vaccination behaviors reported by Linga et al. (31) predicted intention to get vaccinated against seasonal influenza in the United States. The PMT's significant predictors of getting vaccinated were perceived severity, vulnerability, self-efficacy, and response efficacy. Evidence of the applicability of PMT on COVID-19 vaccine hesitancy is scarce. Only a few studies have reported the COVID-19 vaccine hesitancy protection motivation theory. Results showed that severity, self-efficacy, response efficacy, and response cost emerged as significant predictors of COVID-19 vaccine acceptance/hesitancy (32). Further evidence is needed to validate the application of PMT constructs to explain COVID-19 vaccine hesitancy and post-vaccination adherence to safety measures.

These studies have also not focused on psychological constructs influencing reluctance or acceptance toward COVID-19 vaccination. Post-vaccination safety behaviors are also not the focus of research based on vaccine hesitancy. Therefore, the present research is conceptualized with two main objectives. The first objective was *to assess the predictive role of PMT constructs in COVID-19 vaccine hesitancy* and the second objective was *to understand the factors involved in adapting safety behaviors after vaccination*. A mixed-method approach was utilized to achieve these objectives. This approach helped to uncover the social and personal realities through multiple lenses (11, 12). Current research employed the convergence method of the mixed-method approach, which is used when there is a need to understand the research problem by employing both quantitative and qualitative approaches simultaneously (33, 34). This would help in drawing a clear picture of factors associated with vaccine hesitancy and adherence to safety behaviors in the Pakistani context.

## 2. Methods

### 2.1. Sample

For the quantitative part, the sample consisted of 1,736 (58.9% female subjects and 41.1% male subjects) individuals from the vaccine-eligible population [12 years and above, as per criteria of the National Command and Operation Centre (35)] with a mean age of 29 years, mostly unmarried (63.4%), and having an undergraduate education (62.4%) to the criteria. The sample size was calculated using Krejcie and Morgan (36) formula, s = X2NP (1-P) ÷ d2(N1)+X2P(1-P), where s = required sample size, X2 = the table value of chi-square for 1 degree of freedom at the desired confidence level (3.841), *N* = the population size, and *P* = the population proportion. Using this formula, Krejcie and Morgan (36) suggested that for any number of populations over 20,000, a sample size of 384 is sufficient. We have used this approach as the minimum criteria for the required sample size calculation. The sample was selected using the convenience-sampling technique. Most of the sample was vaccinated with at least with one dose (89.6%), never contracted the disease (75.3%), and had close relatives getting infected with COVID-19 (66.4%).

For the qualitative study, the sample consisted of 23 individuals (47.8% female subjects and 52% male subjects) with ages ranging from 13 to 59 years (M = 35.52, SD = 14.14 years). Sampling was performed using a purposive sampling technique in which participants were selected based on vaccination status and willingness to provide data. For further sample characteristics, see Tables 1, 2.

**Table 1:** Sample characteristics (*N* = 1,736).

**Characteristics**	**M(SD)/f (%)**	**Characteristics**	**M(SD)/f (%)**
**Age**	29.00 (12.16)	**Education level**	
**Gender**	Below 12 years	492 (18.3)	
Male	713 (41.1)	13–16 years	1,083 (62.4)
Female	1,023 (58.9)	Above 16 years	161 (9.3)
**Marital status**		**Current work status**	
Unmarried	1,104 (63.4)	Employed	489 (28.2)
Married	585 (33.7)	Student	911 (52.5)
Divorced/widowed	47 (2.7)	Unemployed	117 (6.7)
**Region**		Retired	44 (2.5)
Punjab	935 (53.9)	Housewife	175 (10.1)
Sindh	45 (2.6)	**Average family income**	
Balochistan	24 (1.4)	Below Rs. 30,000	302 (17.4)
Khyber Pakhtunkhwa	135 (7.8)	Between Rs.30,000–50,000	473 (27.2)
Gilgit Baltistan	37 (2.1)	More than Rs. 50,000	961 (55.4)
Islamabad Capital Territory	447 (25.7)	**Area of residence**	
Azad Jammu and Kashmir	113 (6.5)	Urban	1,396 (80.4)
		Rural	340 (19.6)
**Chronic illness**		**Psychological illness**	
Yes	90 (5.2)	Yes	62 (3.6)
No	1,646 (94.8)	No	1,674 (96.4)

F, Frequency; %, Percentage, M; Mean; SD, Standard Deviation.

**Table 2:** Participants characteristics in Phase 2 (*N* = 23).

**Characteristics**	**M (SD)/f (%)**
**Age**	35.52 (14.14)
**Gender**	
Male	12 (52.17)
Female	11 (47.83)
**Marital status**	
Unmarried	10 (43.48)
Married	13 (56.52)
**Education level**	
Below 12 years	03 (13.04)
13–16 years	14 (60.87)
Above 16 years	06 (26.09)
**Current work status**	
Employed	14 (60.87)
Student	07 (30.43)
Housewife	02 (8.70)
**Vaccination status**	
Completely vaccinated	10 (43.48)
Partially vaccinated	12 (52.17)
Vaccinated with booster shots	01 (4.35)
**COVID_19 infection history**	
Infected once	18 (78.26)
Infected twice	01 (4.35)
Never infected	04 (17.39)

f, Frequency; %, Percentage; M, Mean; SD, Standard deviation.

### 2.2. Instrument

For the quantitative part, a self-generated questionnaire was used for this cross-sectional research. The questionnaire was developed and validated (37) in the national language of Pakistan, Urdu, to reduce the language barrier (see Table 3). Therefore, everyone, including those who do not have a strong educational background, can understand and fill out the questionnaire without any difficulty. The comprehensive procedure of scale development was followed to generate the scale involving problem identification, literature review, item generation, expert review, pilot testing, and final scale. The reliability of the scale was within the acceptable range (0.71–0.89). To assess face validity, subject experts were asked to review the items and gauge their suitability and clarity for measuring the variable of interest.

**Table 3:** Items of the protection motivation theory.

**Constructs**	**Items**
Severity	COVID-19 is a deadly disease
	Getting infected with COVID-19 can result in serious health issues One can possibly die from COVID-19
Vulnerability	I am at a constant risk of getting infected with COVID-19
	I am worried about getting infected with COVID-19
	The chances of me getting infected with COVID-19 are higher without getting vaccinated
Rewards	It is important for me to get myself vaccinated against COVID-19
	I can travel around freely after getting vaccinated against COVID-19
	Contracting COVID-19 would be a less of worry for me after getting vaccinated
	I want to get vaccinated because it's free of cost
	It is my social responsibility to get vaccinated against COVID-19
Self-efficacy	It is easy to get vaccinated against COVID-19
	I can choose to get vaccinated against COVID-19 at will
Response efficacy	Getting vaccinated can reduce the risk of getting infected with COVID-19
	Getting vaccinated can help prevent the spread of COVID-19
	Vaccine is the only effective option to prevent oneself from serious effects of COVID-19
Response cost	Getting vaccinated against COVID-19 can cause side effects
	It is time consuming to get vaccinated against COVID-19
	It is painful to get vaccinated against COVID-19

For the qualitative part, semi-structured interviews were conducted to collect the data. This technique of interviews is useful in exploring the meaning behind respondents' experiences through open-ended questions. The interview guide was developed based on the conceptual framework, literature review, and research questions. The interview guide comprised open-ended questions based on the subjective experiences of the respondents. The questions of the interview protocols are as follows:

1. What are the factors that influence your attitude toward safety behaviors?
2. In your opinion, what is the importance of getting a vaccine against COVID-19?
3. Have you been voluntarily vaccinated or forcefully?
4. In your opinion, what are the chances of being re-infected with COVID-19 after vaccination?
5. How much are you aware of the safety behaviors and standard operating procedures (SOPs) to prevent COVID-19 infection?
6. Is it necessary to wear masks, wash hands regularly, and maintain social distancing even after vaccination?
7. Do you wear masks, wash hands regularly, and maintain social distancing even after vaccination?
8. What are the factors that influence your attitude toward safety behaviors?

The researchers also used different probes, such as “*Please elaborate[sic] this point”* and “*Can you explain this further”* to give respondents a chance to fully explain their views.

### 2.3. Procedure and data collection

The data for the quantitative part were collected using the survey method. A total of 2,500 questionnaires were distributed, out of which 1,736 participants responded. The return rate was 69.4%. The consent form, in Urdu, was also distributed along with the questionnaire to obtain the consent of participants for the research. Furthermore, the questionnaire was also forwarded online by generating a link in Google Forms. The link was distributed on different social media platforms, i.e., Facebook, WhatsApp, LinkedIn, and Instagram. However, the focus was kept on collecting data in person to avoid false or untrue responses. Nonetheless, the majority of the data was collected in hard copy.

For the qualitative part, we conducted a qualitative study using a phenomenological approach to evaluate the factors related to COVID-19 safety behaviors. This approach aims to focus on the similar characteristics and personal experiences of the respondents (38). This method is useful for in-depth analysis of factors associated with adherence to safety behaviors to prevent COVID-19 infection. Thematic analysis was used to analyze the data. The reported results followed the Consolidated Criteria for Reporting Qualitative Study (COREQ) checklist. A total of 33 participants were interviewed. The consent form was used to receive data from only those participants who were genuinely willing to participate in this study. Participants were approached by personal and social contacts. Rapport and regular contact were built *via* communication. The average duration of an interview lasted from 20 to 25 min based on the convenience and answers of the respondent. Interviews were recorded with the participant's consent for in-depth analysis.

### 2.4. Analysis scheme

Statistical Package for the Social Sciences, IBM SPSS (Version 23), was used to analyze quantitative data in Phase 1. The statistical procedures that were applied included descriptive statistics, frequencies, mean, reliability analysis, and regression analysis, which are reported in tables. The data acquired through interviews were transcribed. Themes were sorted and proposed inductively without using the pre-existing coding framework as guided by Braun and Clarke (39). Braun and Clarke's (39) six phases were followed for thematic analysis: (1) Initially, the potential themes were identified by re-reading the data from transcriptions and familiarizing with it. (2) The codes were reviewed to retain the themes that were representative of diverse factors to include subthemes. The research questions helped to select the relevant themes for analysis. (3) Theme-relevant quotes were identified. (4) Themes were reviewed again to verify the relevance and representation of the data, leading to a thematic map. (5) Then, the themes were reviewed to define and name them. (6) The write-up was carried out verbatim with themes and sub-themes. This method helped to explore in-depth factors associated with compliance and non-compliance to safety behaviors related to COVID-19 infection.

The reliability of the process was assured by making the research procedure transparent by reporting data collection to data analysis step-by-step. The second and third authors rechecked the generated codes with consensus indicating the good status of the codes. To address conformability, i.e., objectivity, all data were audio-to-text transcribed and analyzed with insightful discussions with all authors to minimize subjectivity and cater to the researchers' reflexivity. Transferability was addressed by gathering a plethora of data and presenting parsimoniously in the Results and Discussion.

### 2.5. Ethical considerations

Informed consent from the participants was taken. The respondents of the study were informed about the anonymity and confidentiality of their information and the correct use of the information obtained through this survey. The participant's right to privacy was also protected. The participants of the study were first elaborated on the purpose of the study to give them insight into the rationale of the research. They were given the right to withdraw from this study at any time. Moreover, enough time was given to participants to respond to each question after carefully understanding the context of the statement written and recording their true responses. The participants were not harmed in any way or form. The biases on the researcher's end were avoided and there was no discrimination made whatsoever while collecting data for this research. In addition, there was no modification of the data collected and only the responses given by participants were used to make further inferences. The study was conducted in accordance with the Declaration of Helsinki. As part of the regular ethical procedures, the study design and data collection procedures were evaluated against the Ethical Decision Tree and approved by the Local Ethics Committee of COMSATS University Islamabad, Pakistan.

## 3. Results

The present study was conceptualized into two phases.

### 3.1. Results of Phase 1

A sample of 1,736 individuals from the vaccine-eligible population (12 years and above) was selected using the convenience-sampling technique from all over Pakistan. The demographic characteristics and COVID-19-related information of the respondents are presented in Table 4. The mean age was 29.00 years (SD = 12.16), 58.9% were female, and 50% of the respondents were students. A total of 75% of respondents were not affected by COVID-19 and almost 90% received the vaccination. Most (78.4%) of the respondents were in favor of getting their younger children/siblings vaccinated against COVID-19 (78.4%), whereas 87.4% were in favor of getting vaccinated against other diseases too.

**Table 4:** COVID-19 vaccine-related sample characteristics (*N* = 1,736).

**COVID-19 related characteristics**	**Yes [*f* (%)]**	**No [*f* (%)]**
Have you ever been infected with COVID-19?	428 (24.7)	1,308 (75.3)
Family member infected with COVID-19?	744 (42.9)	992 (57.1)
Closed relative infected with COVID-19?	1,152 (66.4)	584 (33.6)
Vaccinated against COVID-19	1,555 (89.6)	181 (10.4)
In favor of getting younger children/siblings vaccinated against COVID-19	1,361 (78.4)	375 (21.6)
In favor of getting vaccinated against other diseases	1,518 (87.4)	218 (12.6)

F, Frequency; %, Percentage.

Tables 5–7 reported the results of the multivariable linear regression analyses (stepwise) and examined the independent association of several determinants and the outcomes of interest. In terms of demographic variables as determinants of vaccine hesitancy, the results showed that being male, old age, not vaccinated, and not infected with COVID-19 (themselves and family members) are strongly associated with vaccination hesitancy. Individuals who received the vaccination and were infected with COVID-19 (themselves, family members, and relatives) are considered more vulnerable to the severity of the threat and are more positive toward vaccination.

**Table 5:** Multiple linear regression analysis of determinants and the outcomes of interest (*N* = 1,736).

	**Response cost**				
	**Model 1**	**Model 2**	**Model 3**	**95% Cl**	
**Variables**	**B**	**B**	**B**	**LL**	**UL**
(Constant)	9.414	9.553	9.522	9.104	9.941
Vaccinated	−1.234	−1.195	−1.199	−1.630	−0.767
Family_Member_Infected_with_COVID-19		−0.403	−0.587	−0.887	−0.286
Infected_With_COVID-19			0.454	0.109	0.799
*R* ^2^	0.018	0.023	0.027		
*F*	31.289^***^	20.107^***^	15.671^***^		
Δ*R*^2^		0.005	0.004		
ΔF		8.785	6.667		
	**Severity**				
	**Model 1**	**Model 2**	**Model 3**	**95% Cl**	
**Variables**	**B**	**B**	**B**	**LL**	**UL**
(Constant)	11.824	10.756	10.979	10.541	11.418
Closed_Relative_Infected_With_COVID-19	0.904	0.859	0.805	0.536	1.075
Vaccinated		1.224	1.236	0.822	1.650
Gender (ref. male)			−0.481	−0.739	−0.222
*R* ^2^	0.024	0.043	0.050		
*F*	42.787^***^	38.472^***^	30.267^***^		
Δ*R*^2^		0.018	0.007		
Δ*F*		33.358	13.312		
	**Self-efficacy**				
	**Model 1**	**Model 2**	**Model 3**	**95% Cl**	
**Variables**	**B**	**B**	**B**	**LL**	**UL**
(Constant)	7.149	7.797	7.542	7.182	7.901
Vaccinated	1.022	1.033	0.997	0.711	1.283
Age		−0.023	−0.022	−0.029	−0.015
Closed_Relative_Infected_With_COVID_19			0.404	0.219	0.589
*R* ^2^	0.027	0.048	0.058		
*F*	47.693^***^	43.312^***^	35.254^***^		
Δ*R*^2^		0.021	0.010		
Δ*F*		37.915^***^	18.274^***^		

^***^p < 0.001.

**Table 6:** Multiple linear regression analysis of determinants and the outcomes of interest (*N* = 1,736).

	**Threat appraisal**					
	**Model 1**	**Model 2**	**Model 3**	**Model 4**	**95% Cl**	
**Variables**	**B**	**B**	**B**	**B**	**LL**	**UL**
(Constant)	36.552	35.393	35.355	35.697	34.454	36.939
Vaccinated	4.694	4.522	4.464	4.483	3.308	5.657
Closed_Relative_Infected_With_COVID-19		1.980	1.373	1.300	0.419	2.182
Family_Member_Infected_With_COVID-19			1.149	1.130	0.291	1.970
Gender (ref. male)				−0.737	−1.469	−0.004
*R* ^2^	0.034	0.048	0.052	0.054		
*F*	60.547^***^	43.729^***^	31.656^***^	24.755^***^		
Δ*R*^2^		0.014	0.004	0.002		
Δ*F*		26.547	7.197	3.893		
	**Vulnerability**					
	**Model 1**	**Model 2**	**Model 3**	**Model 4**	**95% Cl**	
**Variables**	**B**	**B**	**B**	**B**	**LL**	**UL**
(Constant)	9.260	8.953	8.923	8.488	8.032	8.945
Family_Member_Infected_With_COVID-19	0.978	0.653	0.456	0.444	0.095	0.793
Closed_Relative_Infected_With_COVID-19		0.672	0.656	0.645	0.309	0.980
Infected_With_COVID-19			0.508	0.505	0.148	0.863
Vaccinated				0.500	0.052	0.948
*R* ^2^	0.026	0.035	0.040	0.042		
*F*	47.614^***^	31.702^***^	23.793^***^	19.082^***^		
Δ*R*^2^		0.009	0.004	0.003		
Δ*F*		15.394	7.730	4.794		

**Table 7:** Multiple linear regression analysis of determinants and the outcomes of interest.

	**Copping appraisal**			
	**Model 1**	**Model 2**	**95% Cl**	
**Variables**	**B**	**B**	**LL**	**UL**
(Constant)	26.315	27.574	26.759	28.389
Vaccinated	1.799	1.820	1.134	2.506
Age		−0.044	−0.061	−0.027
*R* ^2^	0.015	0.029		
*F*	26.089^***^	25.768^***^		
Δ*R*^2^		0.014		
**ΔF**		25.084		
	**Response efficacy**			
	**Model 1**	**Model 2**	**95% Cl**	
**Variables**	**B**	**B**	**LL**	**UL**
(Constant)	9.751	10.322	9.806	10.839
Vaccinated	2.011	2.020	1.585	2.455
Age		−0.020	−0.031	−0.009
*R* ^2^	0.045	0.052		
*F*	81.768^***^	47.596^***^		
Δ*R*^2^		0.007		
Δ*F*		12.864		

### 3.2. Results of Phase 2

For the qualitative part, 23 in-depth interviews were conducted with vaccinated and partially vaccinated individuals from the vaccine-eligible population (12 years and above). The mean age was 35.52 years, 47.8% were female subjects and 52.2% were male subjects, and most were partially vaccinated and affected by COVID-19 infection. From interviews, protocols following major themes were generated.

#### 3.2.1. Lack of knowledge and negative attitudes toward the COVID-19 vaccine

Most of the respondents had a vague and incorrect understanding of how a vaccine functions. According to respondent 1, “*It is important for [sic]lethal virus,[sic] because it is vital not only to keep yourself safe from this, it is also important to keep your family members and surrounding safe*.” Similarly, respondents 2 and 4 stated, “*A vaccine stops the infection in a body and the person won't be harmful anymore*.” Few of them had perceived the effectiveness of vaccine functioning due to their positive or negative post-vaccination experience along with the observation of the vaccine. Respondent 9 stated “*vaccine has no importance as I got the disease even after getting first shot*.” Respondent 14 had similar views saying, “*Vaccine has no as such importance and no vaccine has been invented which is[sic] specifically boost immunity against COVID. However, there is no harm in getting vaccinated*.” Inquiring about their attitude toward vaccination, most of the respondents reported to be forcefully vaccinated and had anti-vaccination attitudes. According to respondent 1, “*I got vaccinated against my will keeping in view the government restrictions*.” Respondent 10 said, “*I got [sic]vaccine shot due to the restriction on entry in[sic] the university without [sic]vaccine card but I am afraid that I[sic] will be bad for my health*.”

#### 3.2.2. Religious beliefs regarding safety behaviors

One of the major causes of non-compliance toward safety behaviors is the religious perception regarding these safety behaviors. As one of the respondents reported, “*It is the will of God that decides whether I live or die, not wearing a mask or keeping [sic]distance from people.”* One of the factors that developed a negative attitude toward these safety behaviors is the restriction on large-scale religious gatherings, especially in the holy month of fasting. One of the respondents said, “*How will people refrain from going to mosques and not offer the evening prayers in Ramazan which is essential before fasting.”*

#### 3.2.3. Fluctuating rates of infections between COVID-19 waves

The compliance to the safety measures (e.g., SOPs) was higher when mortality rate and reported cases of COVID-19 increased. Pakistan had observed four waves of COVID-19 infection where a sharp rise and decline in infection rates had been observed. The data reflect that compliance with safety behaviors correlates with fluctuating rates of reported COVID-19 cases. As one respondent said, “*I usually strictly follow safety behaviors like mask wearing and social distancing when COVID infection rates gets[sic] high or the death rate [sic]are reported to get higher. I feel this is the right attitude since you can't[sic] follow SOPs forever*.” Another respondent reported that “*Logically it is not possible to follow safety behaviors all the time. So, I just look at the situation of [sic]infection rate and adjust my behaviors accordingly. Even authorities also relax restrictions when COVID infection rates decreases[sic].”*

#### 3.2.4. Discomfort due to harsh climate conditions

The findings revealed that safety behaviors, especially wearing masks, are not followed because of discomfort and suffocation due to harsh climate conditions. Pakistan has long and harsh summers in which the temperature increases up to 45–47°C in most of the areas of the country. In such environmental conditions, wearing a mask, which is considered to be mandatory safety behavior in public during the pandemic situation, is a challenge. The data revealed similar findings as respondents mostly complained about similar issues. One of the respondents said “*most people feel suffocated due to wearing masks, especially being students when we are in classrooms full of students, in such an extreme weather we can't[sic] wear masks as practically it is not possible*.” Another respondent reported “*how can we wear masks in [sic]summer time, I feel suffocated*.” One respondent acknowledged, “*mask wearing is the most important safety behavior but practical situations are also there. May be government and pharmaceutical companies should look into manufacturing masks which are according to harsh climates*.”

#### 3.2.5. Lack of strict government regulations

Data show that the lack of strict government regulations is one of the major factors behind the lack of adherence to safety behaviors against the COVID-19 pandemic. A respondent reported, “*But generally in daily life I don't follow safety behaviors like wearing mask[sic] in public place[sic] due to the fact that the authorities don't[sic] care. For example, if you are entering a mall, the officials just check at the entrance if you are wearing a mask or not. Once you are inside, nobody cares. So there is no point in following these safety measures.”* Another respondent said, “*I have seen officials at airports, banks, and malls taking bribes for letting people enter without masks.”* It is also highlighted in the data that in countries where strict rules were applied by the government to follow COVID-19 safety behaviors, people tend to show more adherence to safety measures willingly. One respondent said, “*I had been to a foreign country for some time during this pandemic period when travel restrictions were lifted and I had observed that there were very strict rules and implications of not following safety behaviors in public places. This shows that governments in those countries were very serious regarding [sic]COVID situation. Whereas, in Pakistan, government actions convey a non-serious attitude toward[sic] this grave health emergency. For example, during the 1st year of COVID in 2020, [sic]government relaxed shopping restrictions before [sic]Eid festival. This was insane*.”

#### 3.2.6. Social pressures toward non-compliance with safety behaviors

Another important factor that is found to be associated with adherence to safety behaviors to prevent COVID-19 infection is social pressure. It was observed that people who follow safety behaviors experience negative feedback from relatives, friends, and coworkers. Comments such as “*You are being [sic]coward”* and “*It's[sic] going to affect you more if you wear [sic]mask all the time”* reflect a discouraging attitude toward people who willingly follow safety behaviors to prevent the spread of the disease. It is also reported that following safety behaviors is considered a lack of faith in God. One of the respondents reported, “*My coworker said to me that you don't[sic] have faith in Allah and you trust these masks more.”* Another respondent said “*I keep myself clean by doing wazoo before prayers five times a day. This keeps me safe from COVID. I don't[sic] need these sanitizers as this is merely a[sic] propaganda to increase [sic]sale of these products*.” The most prominent aspect was social distancing in mosques during prayer times. Their peers ridiculed people who willingly offered prayers at home. One of the respondents said, “*My neighbors questioned my stance of not going to mosque due to COVID restrictions and said that this is against religion. I then started going to [sic]mosque to offer my prayers.”* These accounts clearly point to the fact that social pressure is a major contributing factor toward non-compliance to safety behaviors among people.

## 4. Discussion

The adverse effects of the COVID-19 pandemic continue all over the world for the past 3 years. Although many vaccines have been developed to immunize people against the virus, the threat of this serious disease still lingers (40, 41). The current study was aimed at identifying the determinants of COVID-19 vaccine hesitancy and acceptance among the general population of Pakistan along with analyzing the awareness levels of vaccine effectiveness leading to SOP adherence after vaccination. The population of the study included those who were eligible to get vaccinated against COVID-19 at the time of the research, including the general population aged 12 years and above. The primary data were collected using a self-generated scale under the framework of protection motivation theory (PMT) and in-depth interviews.

In Pakistan, generally, people have COVID-19 vaccine hesitancy as there is a prevalence of mistrust in vaccines along with the belief in conspiracy theories about the vaccine. Social media, a widely available source of information, has played a role in spreading false information regarding COVID-19 (42). Narratives such as the vaccine damaging or changing the DNA of individuals has also been prevalent. Another narrative claims that the vaccine will result in the person getting affected with coronavirus rather than making a person immune to it. Besides, religious scholars have played their role in making people fearful of the vaccine and believing it will sterilize all Muslims along with other adverse side effects. These narratives are giving birth to many doubts about the safety and efficacy of the COVID-19 vaccine (43), subsequently leading to low confidence in the vaccine (1). Research highlights the low-level acceptance of the COVID-19 vaccine in many countries in Europe, Africa, and the Middle East (44) due to safety concerns and risks associated with the newly developed vaccine (45, 46). Similar results have reported that vaccine hesitancy has affected COVID-19 vaccination programs worldwide (22).

Gender, age, vaccination status, self-infection, and experience of family members' infection are the strongest predictors of vaccination hesitancy and acceptance in the current study. Elderly male participants are more reluctant whereas a positive attitude toward vaccination can be seen in female participants. Female participants, who either had COVID-19 infection or not, followed SOPs and had a favorable attitude to get vaccinated. The literature highlights that vaccine acceptance is positively associated with COVID-19 knowledge, worry/fear regarding COVID-19, higher income, younger age, and testing negative for COVID-19, whereas females and chronic illness are associated with a low rate of vaccine acceptance (47, 48). Another study highlights that females are associated with vaccine acceptance in countries such as Germany, Russia, France, and Sweden (49). The difference in findings could be impacted by several sociocultural factors. The first important finding is that most of the respondents had vague ideas about how a vaccine functions. People's knowledge, attitudes, and habits on any health behaviors are heavily influenced by their sources of information. A large proportion of respondents with higher educational levels acquired knowledge about COVID-19 from media such as TV and radio, as well as from the internet, which is consistent with previous research (50). Many scientists determined that the vaccine's effectiveness reduced with age, producing lower total body immune responses in persons aged 65 to 85 years than those aged 18 to 55 years (51).

Vaccine hesitancy is associated with a lack of trust in the vaccine's efficacy and safety, as well as unavailability of vaccination and carelessness (52). Few of the study participants had perceived the effectiveness of vaccine functioning due to their positive or negative post-vaccination experience along with the observation of the vaccine, which is coherent with another study carried out by Piraveenan et al. (53). Many religious groups, including Protestants, Catholics, Jews, Muslims, Christians, Amish, Hindus, and Sikhs, have religious reasons for their vaccine apprehension. The biggest hurdle observed in Muslim populations was the presence of porcine or non-halal substances in vaccines (54). Religious organizations were seen as conduits for the spread of false information regarding COVID-19, thus instilling distrust in health professionals and healthcare activities among religious adherents (55). Furthermore, religious meetings and rituals were responsible for the transmission of the coronavirus in other cases because religious devotees disobeyed social distancing instructions (56). According to Waris et al. (57), religious beliefs have been linked to varying degrees of compliance with COVID-19 preventive measures. One cause for this disparity could be the intensity with which religious traditions are followed.

It is necessary to evaluate the state of knowledge, beliefs, and preventive behaviors related to SOP adherence in the post-COVID-19 period, as well as to identify factors impacting post-vaccination preventive practices (58). Therefore, this research focuses on the predictors of SOP adherence post-vaccination. Fear of COVID-19 leads to people adhering to SOPs. Considering the results of the study and according to participants' responses on their knowledge, attitudes, and behaviors, there are some predictors identified that add to the lack of SOP adherence post-vaccination. Among the factors revealed, “lack of sense of fear” is one of the major predictors of reduced SOP adherence after vaccination. It leads them to feel safe from getting COVID-19. Consequently, they do not adhere to SOPs. According to research studies, the COVID-19 vaccine lowers worry as well as anxiety about becoming infected by COVID-19 (59).

Another identified predictor of lack of SOP adherence among the population is “public confidence” reported by the respondents about being protected after receiving one vaccine. In addition to this, participants do not adhere to SOPs after vaccination because of their personal experience of not being re-infected even when they did not follow SOPs. Researchers have discussed that those who cannot be immunized due to comorbidities or who do not develop personal immunity to COVID-19 infection are at risk from vaccine refusers and people who lack SOPs adherence (60).

The following limitations of this study should be kept in mind for future research. Owing to the non-availability of the national database due to security concerns, the random sampling technique was not used for the quantitative part, which affects the generalizability of the research findings. The sample collected mostly consisted of vaccinated individuals. The sample comprised mostly of those whose education level was between 13 and 16 years of education. There was less representation of those with a low educational background and socioeconomic status in the study because it was logistically difficult to collect data from such a diverse group as the population of the study was the general public of Pakistan. The verification of vaccination status was the major limitation in the qualitative part. Future researchers may improve the sampling strategy to collect data that is equally representative of all the education levels and socioeconomic groups of Pakistan. As the findings of this research conclude five major factors contributing to vaccine acceptance and one determining vaccine hesitancy, future research should be focused on social factors beyond the health sector that are contributing to the acceptance of and hesitancy toward COVID-19 vaccines under the framework of protection motivation theory.

## 5. Conclusion

As of January 2023, Pakistan has administered a total of 317,696,373 doses of vaccine, with 56.8% of the population fully vaccinated. The findings of the present study show that effective measures should be taken to address the problems related to vaccine acceptance and all the institutes have to play an effective role to create awareness related to the safety, efficacy, and acceptance of the COVID-19 vaccine. It is also recommended that long-term policy measures should be taken to promote the acceptance of health-related safety behaviors. Programs should be designed for communities to raise awareness of communicable diseases and their prevention.

## Data availability statement

The original contributions presented in the study are included in the article/supplementary material, further inquiries can be directed to the corresponding author.

## Ethics statement

The studies involving human participants were reviewed and approved by COMSATS University Islamabad, Pakistan. Written informed consent to participate in this study was provided by the participants' legal guardian/next of kin.

## Author contributions

AI, AM, SZ, SW, MN, and HK participated in the design of the study and contributed to the data collection, data analysis, and interpretation. AI and AM the principal investigators, designed the study, were responsible for the statistical analysis and interpretation, and wrote the article. SZ wrote the introduction of the article. All authors have read and approved the final version of the manuscript.
